# Mosaic HIV-1 vaccine and SHIV challenge strain V2 loop sequence identity and protection in primates

**DOI:** 10.1038/s41541-024-00974-1

**Published:** 2024-09-30

**Authors:** Kanika Vanshylla, Jeroen Tolboom, Kathryn E. Stephenson, Karin Feddes-de Boer, Annemiek Verwilligen, Sietske Karla Rosendahl Huber, Lucy Rutten, Hanneke Schuitemaker, Roland C. Zahn, Dan H. Barouch, Frank Wegmann

**Affiliations:** 1 Janssen Vaccines & Prevention, Leiden, The Netherlands; 2 Center for Virology and Vaccine Research, Beth Israel Deaconess Medical Center, Boston, MA USA; 3 Ragon Institute of MGH, MIT and Harvard, Cambridge, MA USA; 4 Harvard Medical School, Boston, MA USA

**Keywords:** Vaccines, HIV infections

## Abstract

The failure of human vaccine efficacy trials assessing a mosaic HIV-1 vaccine calls into question the translatability of preclinical SHIV challenge studies that demonstrated high efficacy of this vaccine in primates. Here we present a post hoc immune correlates analysis of HIV-1 Env peptide-binding antibody responses from the NHP13-19 study identifying the V2 loop as the principal correlate of protection in primates. Moreover, we found high V2 loop sequence identity between the Mos1 vaccine component and the SHIV challenge strain, while the vaccine showed considerably lower V2 identity to globally circulating HIV-1 sequences. Thus, the induction of immune responses against the V2 epitope, which had exceptional identity between the vaccine and challenge Env strains, may have contributed to the high protection in primates.

Despite decades of research, an effective HIV-1 vaccine is yet to be achieved. Several prophylactic vaccines have been tested^1,2^, but to date only the RV144/Thai trial, showed any efficacy (31.2%) in the modified intention-to-treat analysis^3^. The high sequence diversity of circulating strains^4^ makes a universal HIV-1 vaccine a difficult goal. A multi-component approach that harnesses cellular and humoral immunity, including broadly neutralizing antibodies that protect against multiple strains, is likely required to achieve sterilizing immunity with an HIV-1 vaccine^1^.

Non-human primates (NHPs) are the preferred animal model for evaluation of HIV-1 vaccine regimens^5,6^, however, they necessitate the use of chimeric simian and HIV-1 viruses (SHIV) that carry the HIV-1 Env in a simian immunodeficiency virus backbone to establish infection. Consequently, the NHP model restricts the assessment of HIV-1 vaccines in challenge settings to the Env component. In an effort to bridge preclinical and clinical outcomes, parallel studies in primates (NHP13-19 study) (Supplementary Fig. 1a) and humans (APPROACH, NCT02935686)^7^ previously tested the mosaic adenovirus serotype 26 (Ad26)-based HIV-1 vaccine candidates in combination with the clade C Env gp140 protein (C97ZA strain). Immunization with Ad26/Ad26+gp140, followed by a series of six consecutive intrarectal viral challenges with the heterologous SHIV-SF162P3 strain, resulted in 67% complete protection from infection^7^. Env-specific ELISA and ELISPOT responses were found to be principal correlates of protection in the NHP13-19 study and similarly robust immunogenicity was observed for the Ad26/Ad26+gp140 regimen in the APPROACH trial^7^. After inclusion of additional clade-specific Ad26-based vaccine components (Mos2S), the mosaic Ad26/Ad26+gp140 vaccine approach was assessed in a Phase 2b efficacy study (Imbokodo, NCT03060629) and a Phase 3 efficacy study (Mosaico, NCT03964415) (Supplementary Fig. 1b). However, vaccination did not prevent HIV-1 infection acquisition in a high-risk population of young women in sub-Saharan Africa (Imbokodo) nor in cisgender men and transgender people (Mosaico)^8^. This outcome was in stark contrast to the high level of efficacy seen in the primate model.

In an effort to understand the basis of the high efficacy from Ad26 vaccine immunization in the NHP13-19 study, we now did a post hoc correlates analyses. To study the Env specificity of serum antibodies, we performed a global HIV-1 peptide microarray using sera from week 56 (W56), collected 4 weeks after the final immunization to assess peak responses (Supplementary Fig. 1a). The microarray measures the binding of serum IgG to linear HIV-1 peptides spanning the Env, Gag and Pol regions and covers the breadth of globally circulating HIV-1 strains^9^. This microarray revealed the Env V2, C2, and V3 domains as prominent antibody target sites (Fig. 1a). Vaccine efficacy was measured as the number of individual SHIV-SF162P3 challenges that animals can withstand without being systemically infected and referred to as time-to-infection (TTI). Accumulated counts of V2 loop peptides bound by serum antibodies at W56 showed the highest degree of correlation with TTI (47%, *P* < 0.001; Spearman correlation) (Fig. 1a and Supplementary Fig. 1c). Amongst the regimens, immune sera from the Ad26/Ad26+gp140 group recognised the highest number of different V2 loop peptides (median 26; Fig. 1b). Correlation of binding magnitude of immune sera to individual peptides with TTI showed V2 specific peptides as the top 8 peptides, accounting for 53% (*n* = 16) of the top 30 peptides with the highest degree of correlation to TTI (Fig. 1c). Dissecting the V2 loop response at the amino acid level revealed a high degree of correlation of TTI to the V2 hotspot, an immunodominant region within the V2 loop identified in the RV144 Trial^10–12^. Peptides including residues 166, 167, 170, and 173 were highly significant predictors of vaccine efficacy in SHIV-challenged primates (Fig. 1d and Supplementary Fig. 1d). The global peptide microarray dataset was integrated with previously examined immune parameters from the original correlates analysis^7^ into an updated ordinal logistic prediction model with stepwise selection of the best correlating parameters. This new prediction model identified V2 antibody responses as the principal correlate of protection, along with the Env ELISPOT response (*P* value < 0.001; Fig. 1e and Supplementary Fig. 1e). The primary safety and immunogenicity endpoint for the parallel human APPROACH trial was W28 (4 weeks after third immunization) and hence microarray peptide-binding analyses of W28 sera from the NHP13-19 study was also performed. Similar to W56, integration of the W28 microarray data into the previous correlates analysis^7^ confirmed V2 binding antibody responses as the principal humoral correlate of protection in NHPs (Supplementary Fig. 1f).

**Fig. 1:**
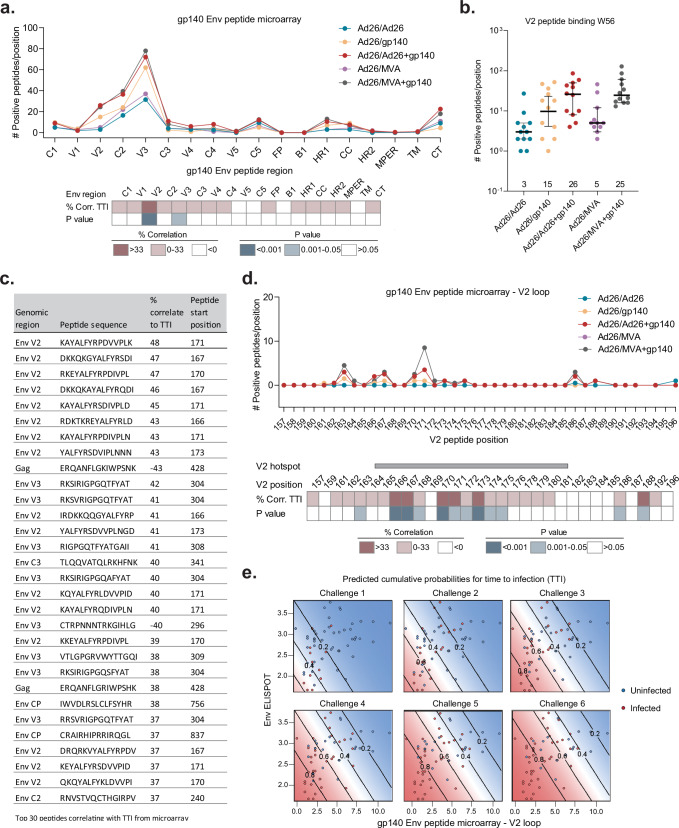
V2 loop responses are highly predictive of time-to-infection in immunized Rhesus monkeys challenged with SHIV. **a** Plot depicting the reactivity of NHP sera from test groups at W56 against HIV-1 Env global peptide array spanning all HIV-1 Env domains. Heatmap below depicts the Spearman correlation of cumulative counts of positive peptide responses with TTI and corresponding *P* values. **b** Dot plot depicting cumulative counts of positive peptide responses against the V2 loop across the test groups at W56. Group medians shown at bottom of graph and bars depict median with 95% CI. **c** Peptide ranking, based on the degree of correlation of individual peptide response magnitude with TTI (in %) at W56. Top 30 peptides along with their genomic region, amino acid sequence and start position are shown. **d** Reactivity of the W56 NHP sera against individual V2 loop peptides. Bar shows residues spanning the V2 hotspot within the V2 loop. Heatmap below depicts the Spearman correlation of magnitude of peptide responses with TTI and corresponding *P* values. **e** Immune correlate-based prediction model showing V2 peptide response (column 1 with no base assay) and Env ELISPOT (column 2 after addition of V2 peptide response as base assay) as the best immune predictors of TTI at W56. Blue and red dots show pooled uninfected and infected monkeys following each challenge timepoint, respectively. Diagonal lines display model-derived probabilities of infection, modeled on V2 microarray and ELISPOT responses. TTI time-to-infection, NHP non-human primate, W week.

Immunogenicity^11^ and sieve analysis^12^ of the RV144 trial previously identified V2 loop-specific IgG as a correlate of reduced infection risk^13,14^ and found that vaccination induced immune pressure on breakthrough virus V2 sequences in humans^12^. With the identification of the V2 loop as the principal correlate of protection in Ad26/Ad26+gp140 immunized primates challenged with SHIV-SF162P3, we decided to compare the V2 loop sequence of SHIV-SF162P3 with those of the vaccines used in the NHP study, namely, Mos1 and C97ZA (Fig. 2a). We included Mos2S in the sequence comparison as the additional component of the tetravalent mosaic Ad26 vaccine used in the Imbokodo and Mosaico trials^15^ (Fig. 2a). Compared with the SHIV-SF162P3 challenge strain, the V2 loop of Mos1 demonstrated the highest sequence identity (76.2%) followed by that of C97ZA (48.9%) and lastly Mos2S (44.0%) (Fig. 2b). Of note, Mos1 covers clade B sequences and thus shows higher identity to the clade B SHIV-SF162P3, whereas both C97ZA and Mos2S cover primarily clade C-based strains (Supplementary Fig. 2a).

**Fig. 2:**
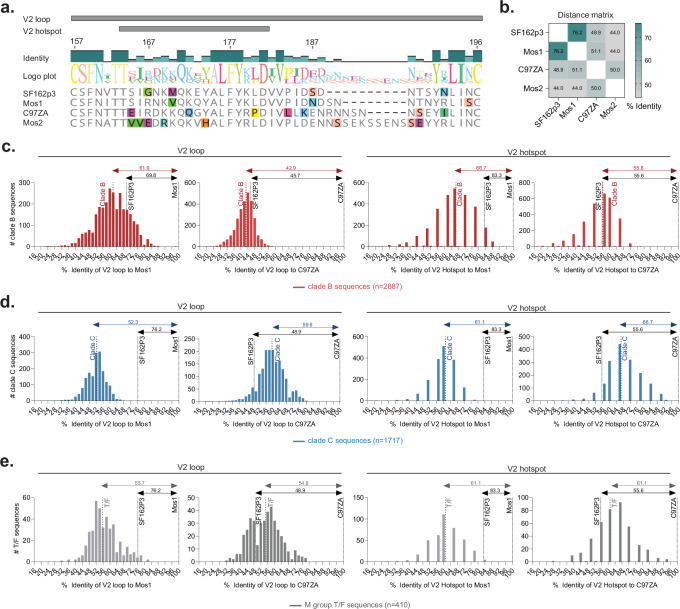
The V2 loop sequence of SHIV-SF162P3 is highly similar to the Mos1 vaccine strain. **a** Env sequence alignment consisting of the V2 loop and the V2 hotspot regions for the NHP challenge strain SF162P3 and vaccine strains, Mos1, Mos2S, and C97ZA. Colors highlight amino acid disagreements within alignment and each color represents a unique amino acid residue. **b** Distance matrix showing % identity of V2 loop sequences shown in (**a**). **c**–**e** frequency distribution of the analyzed circulating clade B (*N* = 2887) (**c**) or clade C (*N* = 1717) (**d**) or M group T/F (*N* = 410) (**e**) strains, based on identity of their V2 loop and V2 hotspot the Mos1 (upper panels) or C97ZA (lower panels) vaccine strains. Black dotted lines depict the SHIV-SF162P3 challenge strain and colored dotted lines represent the circulating strain median in each graph. Numbers on lines above the graphs depict the corresponding % identity to either Mos1 or the C97ZA reference strain. NHP non-human primate, T/F transmitter founder.

To understand the significance of the Mos1 and SHIV-SF162P3 V2 loop sequence identity in the context of a real-world setting, we included in our analysis, *N* = 2887 circulating clade B (Fig. 2c), *N* = 1717 clade C (Fig. 2d) and *N* = 410 transmitter/founder (T/F) sequences (Fig. 2e) from the Los Alamos National Laboratories HIV Sequence database^16^. When looking at the circulating clade B strains, the predominant clade in the Mosaico trial sites^17^, the Mos1 V2 sequence had 69.8% identity with the SHIV-SF162P3 V2 loop, versus 61.9% (range 23–98%) median identity with circulating clade B strains (*N* = 2887) (Fig. 2c). Residues within the V2 hotspot region (positions 166, 167, 170, 173) had shown the highest degree of correlation to TTI (Fig. 1c), and for this region, we observed a high 83.3% identity between Mos1 and SHIV-SF162P3 versus a median 66.7% identity between Mos1 and circulating clade B strains (Fig. 2c). In contrast, the C97ZA clade C vaccine component in the NHP study displayed low identity to both SHIV-SF162P3 and circulating clade B strains in both the V2 loop and V2 hotspot (Fig. 2c). Similarly, the Imbokodo/Mosaico clade C Mos2S vaccine component also displayed low identity to both SHIV-SF162P3 and circulating clade B strains (Supplementary Fig. 2b).

The Imbokodo trial was performed in sub-Saharan Africa, where the predominant circulating strains are clade C^17^. Since Mos1 primarily covers clade B sequences, amino acid sequence analyses comparing V2 loops of Mos1 with *N* = 1717 circulating clade C strains revealed only 52.3% (range 14/71%) median identity versus 76.2% between Mos1 and SHIV-SF162P3 (Fig. 2d). Accordingly, also the V2 hotspot identity of 83.3% between Mos1 and SHIV-SF162P3 was in contrast to the 61.1% identity of Mos1 to circulating clade C strains (Fig. 2d). The clade C components, C97ZA and Mos2S displayed low levels of identity to SHIV-SF162P3 (range 44–55.6%) and moderate identity to circulating clade C strains (range 52–66.7%) in the V2 loop and V2 hotspot regions (Fig. 2d and Supplementary Fig. 2c). This is lower than the similarity seen between the clade B-like Mos1 and SHIV-SF162P3 strains (76.2% for V2 and 83.3% for V2 hotspot), indicating better vaccine-to-challenge strain sequence match in the NHP efficacy study compared with the real-world setting of a human efficacy trial in a clade C region.

Finally, it is widely accepted that a single T/F viral strain results in productive clinical infection in most cases of mucosal HIV-1 transmission^18,19^. Therefore, we looked into *N* = 410 M group T/F-like sequences which included clades A, B, C, D and recombinants. Analysis of T/F-like sequences showed a similar outcome as for the clade B and clade C analysis, with Mos1 showing the highest identity to SHIV-SF162P3 in the V2 loop (76.2%) and V2 hotspot (83.3%) (Fig. 2e and Supplementary Fig. 2d) and median identity of Mos1 to circulating T/F strains being significantly lower, i.e., 55.7% and 61.1% for the V2 loop and V2 hotspot regions, respectively (Fig. 2e). Since residues within the V2 hotspot were the best correlates of protection, we analyzed the T/F viruses for each primary clade i.e., clade C (*n* = 136) and clade B (*n* = 114), and confirmed highest V2 hotspot sequence identity between the Mos1 vaccine component and SHIV-SF162P3 (Supplementary Fig. 2e). Together, the analyses of clade B, clade C and T/F strains all indicate a high vaccine antigen-to-challenge strain V2 sequence identity in the NHP efficacy study compared with the real-world setting of a HIV-1 clinical efficacy trial.

The translatability of vaccine efficacy data observed in animal models to humans can be limited by species-specific differences in absolute quantity and quality of immune biomarkers. To rule out titer differences as a discriminating factor, we performed a quantitative comparison of absolute anti-Env IgG using sera from the NHP20-053 study and immunogenicity data from the ASCENT trial^15^, both of which examined the Mosaico trial vaccine regimen (Supplementary Fig. 2f). The MASCALE (Mass Spectrometry Enabled Conversion to Absolute Levels of ELISA Antibodies) approach^20^ was used to quantify pg/ml amounts of clade C Env binding IgG levels from W28 sera (4 weeks after 3rd immunization) from NHPs and humans. Immunization induced comparable absolute levels of Env-specific IgG in NHPs (median 3.0 × 10^6^ pg/ml) vs. humans (median 1.6 × 10^6^ pg/ml) (Supplementary Fig. 2g), in line with previous observations demonstrating similar immunogenicity in NHPs and humans^7^.

In this post hoc analysis of the NHP13-19 HIV-1 vaccine efficacy study^7^, we found that V2 loop-directed antibody responses were the principal correlate of protection from SHIV infection in primates. Moreover, the V2 loop, and in particular the V2 hotspot region, were highly homologous between the challenge SHIV-SF162P3 strain and Mos1 component of the Ad26/Ad26+gp140 vaccine. Even though the threshold of V2 sequence identity required for protection remains unknown, this high V2 loop sequence homology could be a potential explanation why the primates in the NHP13-19 study showed high (67%) protective efficacy against repeated SHIV challenge^7^. One limitation of this study is the reliance on linear peptides for studying antibody correlates, which does not include conformational antibody binding epitopes that are associated with protection^21^. The diversity of fully characterized SHIV challenge strains available at the time of the NHP13-19 study was limited with SHIV-SF162P3 being one of the few established strains available^5,6^. Moreover, given the high sequence diversity in circulating HIV-1 strains^17^ where an individual may be exposed to highly diverse viruses, the SHIV challenge model would likely not be able to recapitulate this real-world situation. Another difference between animal model and humans is the risk of infection per exposure and total number of exposures. For practicality, the per-exposure infection risk in the NHP model is approximately 1:2 with six controlled exposures. Conversely, the Imbokodo trial enrolled high-risk women in sub-Saharan Africa with a lower estimated per-exposure infection risk^22–25^ but more accumulated exposures predicted during the trial period. Despite these uncertainties, our results provide one probable explanation why high efficacy was observed in the primate study but not in the subsequent Imbokodo and Mosaico human efficacy trials^8^. Thus, there is a need for improvement in current models and for caution in the interpretation of efficacy studies in animal models with a single challenge strain for viruses with high sequence diversity like HIV-1^4^.

## Methods

### NHP13-19 study design and procedures overview

Details of the NHP13-19 study procedures were previously described in the publication from ref. ^7^. In brief, 12 Indian-origin rhesus monkeys (RMs) (*Macaca mulatta*) per group were primed with Ad26.HIV.Mos (5 × 10^10^ viral particles per 0.5 mL) at weeks 0 and 12, and then boosted at weeks 24 and 52 with Ad26.Mos.HIV with or without clade C gp140 protein (C97ZA strain) (250 μg with 425 mcg AdjuPhos), MVA-mosaic (10^8^ plaque-forming units per 0.5 mL) with or without gp140 protein, or gp140 protein alone, all injected intramuscularly. Control group RMs received intramuscular injection of 0.9% saline at weeks 0, 12, 24, and 48. All RMs received intrarectal challenges 1× per week for 6 weeks with 500 50% tissue culture infectious dose (TCID50) of SHIV-SF162P3 starting at study week 76. All procedures were reviewed approved by Beth Israel Deaconess Medical Center Site Institutional Animal Care and Use Committee (IACUC). W28 and W56 samples were used in the immunogenicity analyses.

### ELISA and MASCALE method-based quantitative comparison of NHP vs. human IgG responses against vaccine antigens

For the NHP ELISA, clade C HIV-1 Env C97ZA was directly coated onto 0.5 area high protein binding 96-well OptiPlates (PerkinElmer, catalog#6002520) at 0.5 µg/mL. Wells were washed with wash buffer containing 0.05% Tween-20 in PBS and blocked with block buffer composed of 1% casein in PBS. Plates were subsequently incubated with threefold serial dilutions of NHP samples and reference standard sera using a 1:50 start dilution. Detection was performed using 1:15,000 diluted mouse anti-human IgG-HRP antibody (Jackson, catalog#209-035-098), followed by enhanced chemiluminescent (ECL) substrate (BioRad catalog#170-5061), and luminescence signal was measured in a Synergy plate reader (BioTek) using Gen5^TM^ software version 3.08. A 4-parameter logistic curve fit was used to calculate relative potencies of the samples relative to the standard stock which has an assigned concentration of 10,000 EU/ml. A constant value of 4 was added to the log10-transformed relative potencies to get positive values. The titers were calculated in SAS 9.4 software.

To compare gp140-specific IgG antibody titers quantitatively between NHPs and humans, MASCALE (Mass Spectrometry Enabled Conversion to Absolute Levels of ELISA Antibodies)^20^ was used. The MASCALE method uses levels of representative IgG peptides that are determined by quantitative Mass Spectometry (qMS) and serve as a surrogate for the molar IgG content of a sample, allowing the conversion of responses from arbitrary values of EU/ml measured in ELISA to absolute amounts in picograms (pg). Since the NHP and human ELISAs use different protocols, a dilution series of both NHP and human standards were tested in the NHP ELISA format for quantification by MS. The pg amounts of human STD measured by qMS in the NHP ELISA format were correlated with the EU/ml of human STD in the human ELISA format to derive a conversion formula for human serum samples. The measurement of the human STD in qMS in the NHP ELISA format is done based on the assumptions that, (i) both the NHP and human ELISA methods are linear within the quantifiable range of the respective assays and, (ii) that although binding conditions between NHP and human ELISA differ, the reference amount of human standard present in the assay and its relative correlation to human ELISA EU/ml is maintained between the two assay formats.

The MASCALE method was performed as previously described^20^. Briefly, human and NHP reference standard serum were added to antigen-coated ELISA plates as described above in the NHP ELISA protocol, and buffer-only plates were spiked with synthetic peptides (JPT Peptide Technologies) for generating the mass spectrometry (MS) calibration curve. Samples were prepared by filter-assisted sample preparation, reduction and alkylation followed by tryptic digestion into peptides and stored at −80 °C until solid-phase extraction and analysis by Liquid chromatography with tandem mass spectrometry on a LC-30AD system (Shimadzu) with a 2.1 × 50 mm (1.7 µm) Acquity Ultra Performance Liquid Chromatography BEH300 C18 column (Waters Corporation) at a flow rate of 0.4 mL/min using a LC-30 ACMP autosampler (Shimadzu Nexera). The liquid chromatography system was coupled to a QTRAP 6500+ Triple Quad mass spectrometer (Sciex) operating in the multiple reaction monitoring (MRM) positive ionization mode. Analyst software v1.7 was used for LC-MS/MS acquisition. Data processing was performed using SciexOS v2.1.6 software. Data analysis was performed using JMP software (SAS). To enable quantification of human IgG antibodies, two peptides were selected, VVSVLTVLHQDWLNGK (M 1808.09 gmol^−1^) for human IgG1, 3 and 4 as well as VVSVLTVVHQDWLNGK (M 1794.06 gmol^−1^) for human IgG2. Quantities were obtained in pg peptide (representative of IgG1, 3, 4, and IgG2) per sample by back-calculation of the peak area ratio onto the peptide calibration curve. The amount of each peptide measured by MS was then converted to moles of peptide, followed by conversion to the mass of IgG1, 3, 4 and IgG2, then summed to obtain the total amount of IgG per well. To enable the quantification of NHP IgG, another representative peptide was identified (VVSVLTVTHQDWLNGK, M 1769.036 gmol^−1^). This peptide was universally present in macaque IgG1, 2, 3, and 4, allowing its use as a surrogate peptide to quantify all IgG types for NHP samples. Values below the quantifiable range of the ELISA were excluded from analysis. A linear regression analysis provided the conversion formula for arbitrary ELISA units (EU/mL) to absolute quantities (pg/well). STD curve EU/ml assignment were based on NHP assay dilution factor and derived from the original assigned STD stock concentrations of 100,000 EU/ml and 10,000 EU/ml for the human and NHP STD, respectively.

Conversion formulas generated from NHP and human STD curves for pg/well IgG titer calculation,

NHP formula:

$${\rm{Log}}10{\rm{IgG}}\,({\rm{pg}}/{\rm{well}}\; {\rm{in}}\; {\rm{NHP}}\; {\rm{ELISA}}\; {\rm{format}}\; {\rm{qMS}})=0.878\,{\rm{x}}\mathrm{log}10({\rm{EU}}/{\rm{ml}}\; {\rm{in}}\; {\rm{well}})+1.928$$

Human formula:

$${\rm{Log}}10{\rm{IgG}}({\rm{pg}}/{\rm{well}})=0.883\,{\rm{x}}\mathrm{log}10({\rm{EU}}/{\rm{mL\; in\; well}})+0.566$$

The pg/well was converted into pg/ml serum by considering the minimum required dilution of the assay (1:50) and the amount of sample (1 µL) added to the well. The final pg/ml values were plotted using GraphPad Prism Version 9.5.0.

### NHP20-053 study

For the quantitative analysis of HIV-1 Env IgG titers, serum samples from the NHP20-053 study were used. 10 Indian-origin rhesus monkeys (RMs) (Macaca mulatta) were primed with Ad26.Mos4.HIV (5 × 10^10^ viral particles) at weeks 0 and 12, and then boosted at weeks 24 with Ad26.Mos4.HIV and Clade C gp140 (C97ZA strain) plus mosaic gp140 (125 mcg of each protein) adjuvanted with 425 g AdjuPhos. All immunizations were done via the intramuscular route. All procedures were reviewed approved by BIOQUAL Site IACUC. W28 serum samples were used for immunogenicity analysis via MASCALE.

### ASCENT study

The safety and immunogenicity of the ASCENT trial, a randomized, double-blind, placebo-controlled, Phase 1/2a study (ClinicalTrials.gov NCT02935686) has been previously described^15^. Briefly, participants were administered Ad26.Mos4.HIV or placebo intramuscularly at day 0 and week 12; at weeks 24 and 48, Ad26.Mos4.HIV with Clade C gp140 (C97ZA strain) plus mosaic gp140 (125 mcg of each protein) adjuvanted with 425 g AdjuPhos (group 2b). Study had been conducted in accordance with the Declaration of Helsinki, Good Clinical Practices, and applicable regulatory guidelines with the study protocol being approved by institutional review boards at each study site and carried out with informed consent from participants. Within the scope of the present study, the MASCALE-based quantification of the human standard was performed, and using the regression formula generated, absolute quantities of IgG were computed from previously available binding antibody data^15^ for 90 serum samples from group 2b.

### Global HIV-1 peptide microarray analysis

The global HIV-1 peptide microarray was performed as previously described^9^ to assess antibody responses against linear HIV-1 Env, Gag and Pol peptides. In brief, PepStar peptide microarrays were produced by JPT Peptide Technologies GmbH (Berlin, Germany). Peptides were synthesized on cellulose membranes using the SPOT synthesis technology. Peptide microarrays were produced using a non-contact high-performance microarray printer on epoxy-modified slides (PolyAn; Germany) and stored at 4 °C until use. Microarray slides were incubated with serum at a dilution of 1:200 for 1 h at 30 °C, washed five times with wash buffer (TBS-Buffer + 0.1%Tween-20). Slides incubated with Alexa Fluor 647-conjugated AffiniPure Mouse Anti-Human IgG (H + L) (Jackson ImmunoResearch Laboratories) for 1 h, washed and dried. Secondary antibody alone was used as control. Slides were scanned with a GenePix 4300 A scanner (Molecular Devices), using 635 nm and 532 nm lasers at 500 PMT and 100 Power setting. The fluorescent intensity for each feature (peptide spot) was calculated using GenePix Pro 7 software and GenePix Array List file and the analysis created a GenePix Results file. Mean fluorescent intensity across the triplicate sub-arrays was calculated for each feature custom-designed R script software package. Data was saved as a comma delimited DAT file usable in Excel. The threshold value used to define a minimum positive fluorescent intensity was calculated for each slide using the computational tool rapmad and a custom-designed R script^9^. The raw magnitude, or fluorescent intensity, of antibody binding to individual peptides was sorted and categorized by HIV-1 protein and amino acid start position as aligned to HXB2 HIV-1 reference strain, using a custom-designed R script. The fluorescent intensity of antibody binding on the control slide was subtracted from that of the sample slide. All corrected fluorescent intensities were compared with the calculated threshold for positivity, and all values above the threshold were considered positive. Final graphs were plotted using GraphPad Prism version 9.5.0.

### Statistical analyses

For the microarray peptide assay, Spearman correlations were calculated with time-to-infection. The correlation percentage indicates scaling the correlation values by a factor of 1/100. To assess immune correlates of protection for W56, assays including the microarray peptide (Supplementary Fig. 1), were selected stepwise by cumulative logistic regression on time-to-infection using groups Ad26/Ad26, Ad26/gp140, Ad26/Ad26+gp140, Ad26/MVA and Ad26/MVA+gp140. As described earlier^7^, for W28, the immune correlates prediction model was more powerful without the groups boosted with MVA, and thus MVA-boosted groups were excluded. Selection continued until no assay significantly improved the model, defined as p < 0·05. The placebo does not induce an immune response and hence to avoid bias, the placebo was excluded from all correlate analyses.

### Sequence analyses of SHIV, vaccine component, and circulating HIV-1 strains

Env sequences from SHIV-SF162P3 and the vaccine strains, Mos1 (clade B-based Mosaic), C97ZA (clade C) and Mos2S (clade C-based Mosaic) were used for analysis, along with circulating strain sequences from the Los Alamos National Laboratories HIV Sequence database^16^. For retrieving the Clade C and Clade B sequences, latest premade web alignment (year 2021) of M group without recombinants (A-L) HIV-1 Env amino acid sequences were used where one sequence per patient is included, very similar sequences have been deleted and problematic sequences are removed as per the Los Alamos National Laboratories HIV-1 Sequence database. The 2021 alignments in the database contain sequences published through the end of 2021 (sampling years ranging from 1979–2021). For retrieving T/F, all M group HIV-1 Env sequences acquired up to 100 days post infection (<100 days selection in search field “days from infection”) were assessed, and in this case as well, one sequence per patient was included and problematic sequences removed. Sequences were analyzed using the Geneious Prime® 2022.1.1 software. Multiple sequence alignments were built using the in-built Clustal Omega method to group sequences by similarity. The multiple sequence alignment tests three or more biological sequences of similar length by comparing each sequence against all other sequences within the sequence set. The percent identity is calculated as the number of matches in the alignment row relative to the alignment length. The amino acid alignment and sequence gaps per position are dependent on the sequences used in the multiple sequence alignment. Therefore, depending on the input sequences used within a multiple sequence alignment, the % identity between two given sequences can vary across different multiple sequence alignments. % Identity calculated by the alignment procedure are shown in Fig. 2b, Fig. 2f–h and Supplementary Fig. 2b–d. The following Env regions were used in the analysis: V2 loop with HXb2 amino acid numbering 157–196 and V2 Hotspot with HXb2 amino acid numbering 164–181. Histogram plots of outputs of % identity were generated using the GraphPad Prism version 9.5.0 software.

## Supplementary information

Supplemental Information

## Data Availability

All final data has been included in main figures or supplementary information. Any requests for protocols and reagents should be directed to the corresponding author to be fulfilled under reasonable request.
