# Supplementary material for: Mosaic HIV-1 vaccine and SHIV challenge strain V2 loop sequence identity and protection in primates
Source: NPJ Vaccines. 2024 Sep 30;9:179. doi: 10.1038/s41541-024-00974-1 (PMC11442979; doi:10.1038/s41541-024-00974-1)
Supplement: Supplementary file 1 — Supplemental Information [file 41541_2024_974_MOESM1_ESM.pdf]

Supplementary figure 1

a.

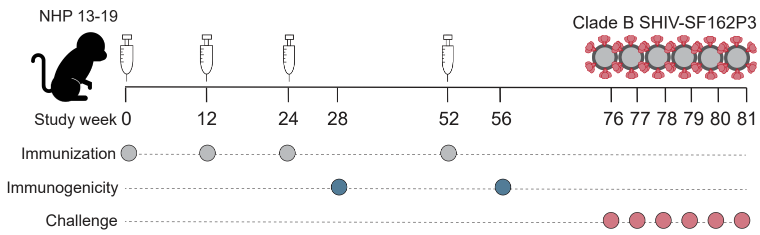

Grp.	N	Week 0	Week 12	Week 24	Week 52	Week 76-81
1	12	Ad26.Mos.HIV	Ad26.Mos.HIV	Ad26.Mos.HIV	Ad26.Mos.HIV	6x i.r. SHIV-SF162P3 challenge weekly
2	12	Ad26.Mos.HIV	Ad26.Mos.HIV	Clade C gp140	Clade C gp140	
3	12	Ad26.Mos.HIV	Ad26.Mos.HIV	Ad26.Mos.HIV + Clade C gp140	Ad26.Mos.HIV + Clade C gp140	
4	12	Ad26.Mos.HIV	Ad26.Mos.HIV	MVA-Mosaic	MVA-Mosaic	
5	12	Ad26.Mos.HIV	Ad26.Mos.HIV	MVA-Mosaic + Clade C gp140	MVA-Mosaic + Clade C gp140	
6	12	Placebo	Placebo	Placebo	Placebo	

Ad26.Mos.HIV = Ad26.Mos1.Gag-Pol + Ad26.Mos2.Gag-Pol + Ad26.Mos1.Env (5x1010 vp total)

Clade C gp140 = 250 mcg protein with 425 mcg AdjuPhos

Placebo = Saline

Group nomenclature used in main figures 1a, 1b and 1d:

Grp. 1 =	Ad26/Ad26	Grp. 1 = Ad26/MVA
Grp. 2 =	Ad26/gp140	Grp. 2 = Ad26/MVA+gp140
Grp. 3 =	Ad26/Ad26+gp140	

b.

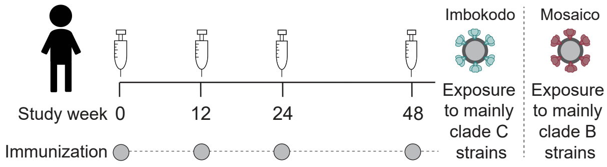

Imbokodo schedule

Grp.	N	Week 0	Week 12	Week 24	Week 48
1	1300	Ad26.Mos4.HIV	Ad26.Mos4.HIV	Ad26.Mos4.HIV + Clade C gp140	Ad26.Mos4.HIV + Clade C gp140
2	1300	Placebo	Placebo	Placebo	Placebo

Mosaico schedule

Grp.	N	Week 0	Week 12	Week 24	Week 48
1	1900	Ad26.Mos4.HIV	Ad26.Mos4.HIV	Ad26.Mos4.HIV + Clade C gp140 + Mosaic gp140	Ad26.Mos4.HIV + Clade C gp140 + Mosaic gp140
2	1900	Placebo	Placebo	Placebo	Placebo

Ad26.Mos4.HIV = Ad26.Mos1.Gag-Pol + Ad26.Mos2.Gag-Pol + Ad26.Mos1.Env + Ad26.Mos2S.Env (5x1010 vp total)

Clade C gp140 = 250 mcg protein with 425 mcg AdjuPhos

Clade C gp140 + Mosaic gp140 = 125 mcg each protein with 425 mcg AdjuPhos

Placebo = Saline

c.

Env region	% Correlation with TTI	P value
C1	8	0.567
V1	16	0.213
V2	47	0.000
C2	23	0.076
V3	30	0.021
C3	25	0.052
V4	19	0.152
C4	12	0.350
V5	-5	0.714
C5	-14	0.285
FP	14	0.289
B1	-19	0.141
HR1	21	0.110
CC	10	0.435
HR2	12	0.342
MPER	18	0.181
TM	-14	0.274
CT	21	0.104

d.

V2 Env region	% Correlation with TTI	P value
157	5	0.717
159	0	0.999
161	12	0.371
162	15	0.249
163	33	0.010
164	15	0.246
165	12	0.344
166	53	0.000
167	51	0.000
168	28	0.028
169	-9	0.483
170	46	0.000
171	43	0.001
172	28	0.029
173	47	0.000
174	27	0.038
175	28	0.027
176	1	0.928
178	14	0.286
179	10	0.438
180	16	0.218
181	-6	0.662
182	-13	0.330
183	-12	0.378
184	-5	0.697
185	-1	0.965
186	25	0.049
187	-7	0.571
188	36	0.005
192	10	0.428
196	-5	0.701

e.

Base Assay:none	
**Test Assay**	**P Value**
A244_V1V2_W56	0.047 *
ADCC_CladeC_W56	0.010 *
ADCC_Mosaic_W56	0.143
ADCD_CladeC_W56	0.382
ADCD_Mosaic_W56	0.100
ADCP_CladeC_W56	0.113
ADCP_Mosaic_W56	0.823
ADNP_CladeC_W56	0.008 **
ADNP_Mosaic_W56	0.003 **
C97ZA_Env_ELISA_W56	0.006 **
CD4_Env_IFN_ICS_W61	0.121
CD8_Env_IFN_ICS_W61	0.979
CN54_V1V2_W56	0.089
Clade_B_case_A2_V1V2_W56	0.006 **
Consensus_CladeC_V1V2_W56	0.122
Mos_Env_ELISA_W56	0.013 *
PTE_Env_ELISPOT_W54	0.001 **
PepArray_gp140_V2_W56	0.000 ***
SF162p3_V1V2_W56	0.032 *

Base Assay:Pep_gp140_V2_W56	
**Test Assay**	**P Value**
A244_V1V2_W56	0.988
ADCC_CladeC_W56	0.684
ADCC_Mosaic_W56	0.540
ADCD_CladeC_W56	0.309
ADCD_Mosaic_W56	0.602
ADCP_CladeC_W56	0.630
ADCP_Mosaic_W56	0.630
ADNP_CladeC_W56	0.635
ADNP_Mosaic_W56	0.215
C97ZA_Env_ELISA_56	0.407
CD4_Env_IFN_ICS_W61	0.721
CD8_Env_IFN_ICS_W61	0.560
CN54_V1V2_W56	0.878
Clade_B_case_A2_V1V2_W56	0.965
Consensus_CladeC_V1V2_W56	0.382
Mos_Env_ELISA_W56	0.406
PTE_Env_ELISPOT_W54	0.044 *
PepArray_gp140_V2_W56	.
SF162p3_V1V2_W56	0.863

f.

Base Assay:none	
Test Assay	P Value
C97ZA_Env_ELISA_W28	0.003 **
PTE_Env_ELISPOT_W26	0.002 **
PepArray_gp140_V1_W28	0.177
PepArray_gp140_V2_W28	0.001 ***
PepArray_gp140_V3_W28	0.017 *

Base Assay:Pep_gp140_V2_W28	
Test Assay	P Value
C97ZA_Env_ELISA_W28	0.106
PTE_Env_ELISPOT_W26	0.001 **
PepArray_gp140_V1_W28	0.827
PepArray_gp140_V2_W28	.
PepArray_gp140_V3_W28	0.635

Supplementary figure 1: Study design for NHP study and human clinical trials and the statistical immune correlates model to predict time-to-infection after SHIV challenge.

a, Schematic showing the study design, grouping, immunization and challenge scheme of the NHP13-19 study. **b**, Schematic of study design for the in-human Phase 2b Imbokodo trial and Phase 3 Mosaico trial. **c**, P values from the spearman correlation analysis of HIV-1 Env peptide array response magnitude vs. TTI at W56. **d**, P values from the spearman correlation analysis of HIV-1 V2 loop peptide array response magnitude vs. TTI at W56. **e**, P values derived from humoral and cellular immune assays at W56 applied to a prediction model for time-to-infection showing V2 peptide responses (column 1 with no base assay) and Env ELISPOT (column 2 after addition of V2 peptide response as base assay) as the best immune predictors of TTI. **f**, P values derived from humoral and cellular immune assays at W28 applied to a prediction model for time-to-infection also confirming showing V2 peptide responses (column 1 with no base assay) and Env ELISPOT (column 2 after addition of V2 peptide response as base assay) as the best immune predictors of TTI.. TTI: time-to-infection; NHP: Non-human primate; W: Week.

Supplementary figure 2

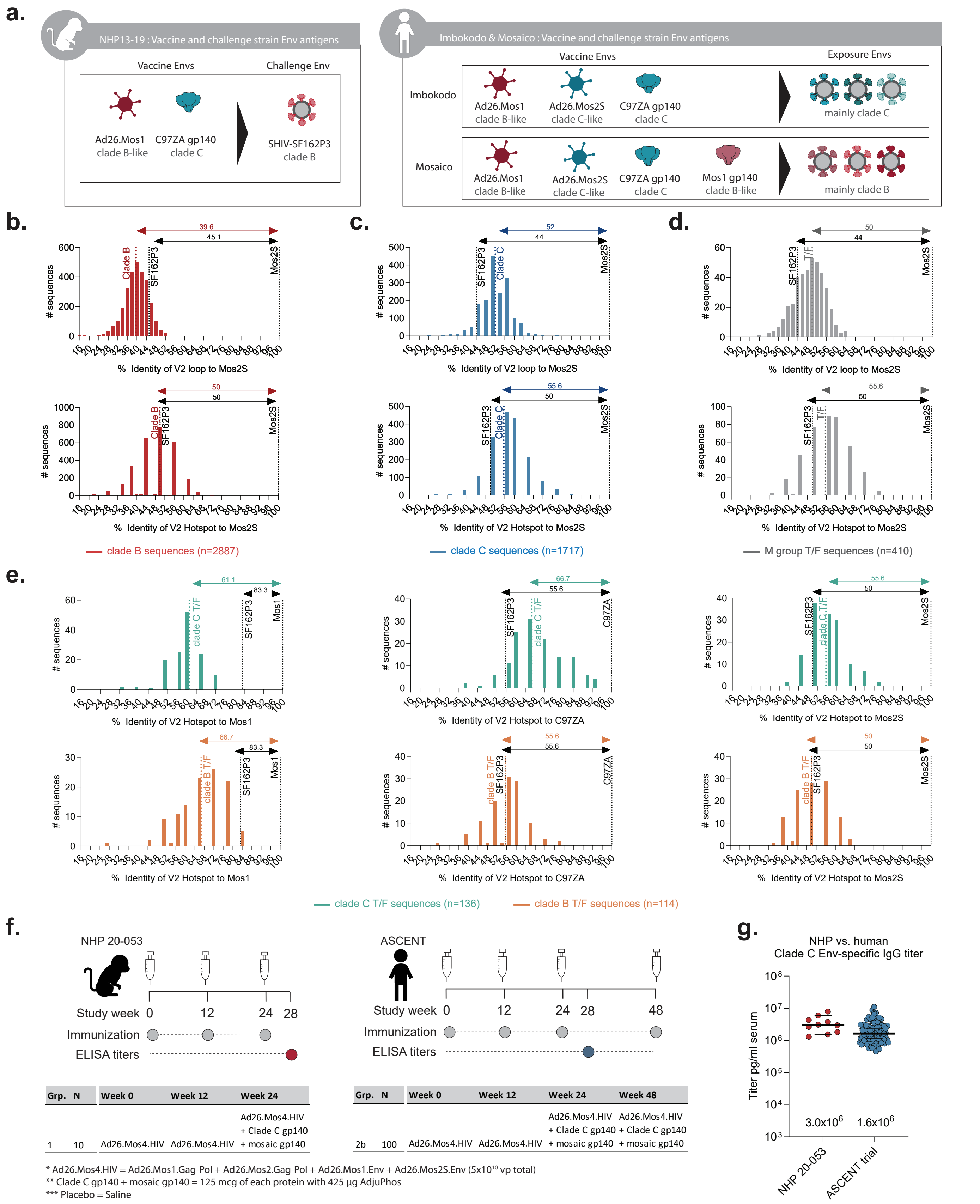

Supplementary Figure 2: Sequence identity between vaccine strains and SHIV challenge strain and comparison of anti-HIV-1 titers in humans vs. NHPs. **a,** Schematic of the Env vaccine components in the NHP13/19 study and the Imbokodo trial. **b-d**, distance of (b) circulating clade C strains (N=1717) (c) circulating clade C strains (N=1717) (d) or circulating M group T/F strains (N=410) along with the SHIV-SF162P3 challenge strain, relative to the Mos2S vaccine sequence in Env regions: V2 loop, V2 hotspot. Black dotted lines depicting the SHIV SF162P3 challenge strain and the colored dotted lines present the circulating strain median in each respective graph. Numbers on the lines above the graph depict the corresponding % identity to either Mos1 or the C97ZA reference strain. **e,** distance of circulating clade C (n=136) and clade B (n=114) subsets of T/F and strains along with the SHIV-SF162P3 challenge strain, relative to the vaccine V2 hotspot. Black dotted lines depicting the SHIV SF162P3 challenge strain and the colored dotted lines present the circulating strain median in each respective graph. Numbers on the lines above the graph depict the corresponding % identity to vaccine strain. **f**, Study schematics of groups used from NHP study 20-053 and the Phase 2a ASCENT trial to compare titers post immunization. **g**, MASCALE-based absolute quantification of clade C C97ZA HIV-1 Env specific IgG titers in NHPs (n=10 from group 1) and humans (n=90 from group 2b) from W28 from studies shown in panel e. Bars show median with 95% CI. Numbers at the bottom of the graph show the median value. MASCALE: Mass Spectrometry Enabled Conversion to Absolute Levels of ELISA Antibodies.
